# The Effect of Levothyroxine Discontinuation Timing on Postoperative Hypothyroidism after Hemithyroidectomy for Papillary Thyroid Microcarcinoma

**DOI:** 10.1155/2016/3240727

**Published:** 2016-05-11

**Authors:** Tae Kwun Ha, Dong Wook Kim, Ha Kyoung Park, Jin Wook Baek, Yoo Jin Lee, Young Mi Park, Do Hun Kim, Soo Jin Jung, Ki Jung Ahn

**Affiliations:** ^1^Department of General Surgery, Busan Paik Hospital, Inje University College of Medicine, Busan 614-735, Republic of Korea; ^2^Department of Radiology, Busan Paik Hospital, Inje University College of Medicine, Busan 614-735, Republic of Korea; ^3^Department of Otorhinolaryngology-Head and Neck Surgery, Busan Paik Hospital, Inje University College of Medicine, Busan 614-735, Republic of Korea; ^4^Department of Pathology, Busan Paik Hospital, Inje University College of Medicine, Busan 614-735, Republic of Korea; ^5^Department of Radiation Oncology, Busan Paik Hospital, Inje University College of Medicine, Busan 614-735, Republic of Korea

## Abstract

*Objective*. No previous studies regarding the appropriate timing of thyroid hormone discontinuation after hemithyroidectomy have been published. This study aimed to identify the appropriate timing for levothyroxine discontinuation after hemithyroidectomy among patients with papillary thyroid microcarcinoma (PTMC).* Methods*. This study retrospectively evaluated 304 patients who underwent ≥1 attempt to discontinue levothyroxine after hemithyroidectomy for treating PTMC between January 2008 and December 2013. Fifty-three patients were excluded because of preoperative hypothyroidism or hyperthyroidism, a history of thyroid hormone or antithyroid therapy, no available serological data, or a postoperative follow-up of <24 months. We evaluated the associations of successful levothyroxine discontinuation with patient age, sex, preoperative serological data, underlying thyroid gland histopathology, anteroposterior diameter of the residual thyroid gland, number of discontinuation attempts, and initial discontinuation timing.* Results*. Among the 251 included patients, 125 patients (49.8%) achieved successful levothyroxine discontinuation during the follow-up period after hemithyroidectomy. There was a significant difference in the outcomes for patients who underwent an initial discontinuation attempt at ≤3 months and ≥4 months after hemithyroidectomy (*p* < 0.001). There were significant differences in the discontinuation outcomes according to underlying thyroid histopathology (*p* = 0.001), preoperative thyroid-stimulating hormone levels (*p* < 0.001), and number of discontinuation attempts (*p* < 0.001).* Conclusions*. Among patients with PTMC, the initial levothyroxine discontinuation attempt is recommended at ≥4 months after hemithyroidectomy.

## 1. Introduction

Papillary thyroid carcinoma is the most common type of thyroid cancer and progresses gradually [1]. A good prognosis can be expected, as surgical treatments for papillary thyroid carcinoma provide positive outcomes [2]. Previous studies have compared total thyroidectomy and hemithyroidectomy for treating papillary thyroid microcarcinoma (PTMC) and found no particular advantage for total thyroidectomy over hemithyroidectomy [3]. Furthermore, hemithyroidectomy can prevent hypoparathyroidism, which is a major complication of total thyroidectomy [4], and reduce the need for postoperative thyroid hormone replacement, depending on the residual thyroid function [5]. However, hemithyroidectomy is generally recommended for low-risk patients with PTMC, who have a single lesion that is restricted to the thyroid gland and no cervical lymph node metastasis [6].

Suppressing thyroid-stimulating hormone (TSH) levels to ≤0.1 mIU/L might enhance the prognosis among high-risk patients with differentiated thyroid cancer, but it does not affect their disease-free survival [7]. Thus, conservative surgery that preserves the thyroid gland should be considered to avoid potential adverse reactions that are associated with TSH suppression [8], because a single lobe contains enough thyroid follicular cells to sustain normal body functions [9]. Therefore, continuous administration of thyroid hormones might not be necessary, unless other factors indicate that thyroid function will be compromised.

Research has continued to evaluate the risk factors for posthemithyroidectomy hypothyroidism, which necessitates thyroid hormone replacement therapy. However, to the best of our knowledge, there are no studies regarding the appropriate timing of thyroid hormone discontinuation after hemithyroidectomy among patients with PTMC. Therefore, we investigated the preoperative and postoperative thyroid functions among patients who underwent hemithyroidectomy for PTMC and compared the associations between levothyroxine discontinuation outcomes and the timing of the discontinuation and other factors.

## 2. Material and Methods

### 2.1. Study Population

Our institutional review board approved this retrospective study and waived the need for informed consent based on the retrospective design. This study reviewed the medical records of 304 patients (284 women and 20 men; age range, 17–71 years; mean age, 42.4 ± 8.4 years) who underwent hemithyroidectomy to treat PTMC and at least one attempt of levothyroxine discontinuation in our hospital between January 2008 and December 2013. The indications for hemithyroidectomy included the absence of prior head and neck radiation, cervical lymph node metastasis, distant metastasis, extrathyroidal extension, and aggressive variants. The exclusion criteria included preoperative hypothyroidism or hyperthyroidism (*n* = 13), a history of thyroid hormone or antithyroid drug treatment (*n* = 10), no available serological data (*n* = 9), and a postoperative follow-up of <24 months (*n* = 21). Thus, we included 251 patients in the analysis (231 women and 20 men; age range, 17–71 years; mean age, 42.4 ± 10.4 years).

### 2.2. Serological and Sonographic Evaluations

The thyroid hormones that we evaluated were free T4 (reference range [RR]: 0.93–1.71 ng/dL), TSH (RR: 0.27–4.20 mIU/L), and thyroglobulin (TG; RR: 1.4–78 ng/dL) during the 7 days before the hemithyroidectomy. These items were also frequently measured after the hemithyroidectomy. Anti-thyroid peroxidase (anti-TPO) antibodies (RR: <5.61 IU/mL) and anti-TG antibodies (RR: <4.11 IU/mL) were measured before surgery, and positive results were defined as anti-TPO levels of ≥5.61 IU/mL or anti-TG levels of ≥4.11 IU/mL. A patient with at least one positive antibody result was considered positive for thyroid autoantibodies. All serological parameters were evaluated using electrochemiluminescence immunoassays with the Elecsys® automatic system (Roche Diagnostics Deutschland GmbH, Mannheim, Germany).

Preoperative ultrasonography was performed using a high-resolution ultrasound scanner (HDI 5000 or iU 22; Phillips Medical Systems, Bothell, WA, USA) with a 5–12 MHz linear probe. The anteroposterior diameter of the residual thyroid gland was retrospectively measured using a picture archiving and communication system.

### 2.3. Hemithyroidectomy and Histopathology

By two surgeons who treat >100 patients with PTMC each year, hemithyroidectomy was performed to preserve the isthmus and contralateral lobe of the thyroid. Among the 251 patients, 153 patients underwent conventional open hemithyroidectomy and 98 patients underwent endoscopic hemithyroidectomy. After the hemithyroidectomy, the histopathological analyses were performed by one pathologist who had 15 years of experience diagnosing thyroid cancer. Based on the histopathological findings, the thyroid glands were classified as normal thyroid, Hashimoto thyroiditis, non-Hashimoto lymphocytic thyroiditis (i.e., diffuse infiltration of lymphocytes and other inflammatory cells, but no typical histopathological features of Hashimoto thyroiditis), Graves' disease, or diffuse hyperplasia [10].

### 2.4. Levothyroxine Initiation and Discontinuation

Based on the preoperative thyroid function tests, all 251 patients exhibited a normal thyroid function. Most patients received 0.15–0.1 mg of levothyroxine sodium after the hemithyroidectomy, although some patients did not receive levothyroxine immediately after the surgery. During the first 12 months after the hemithyroidectomy, the patients' serum TSH levels were measured during postoperative weeks 2–6 and then every 3 months.

Levothyroxine discontinuation was initially attempted after normal thyroid function was confirmed by the thyroid function test results. Levothyroxine administration was restarted if hypothyroidism was detected after the initial levothyroxine discontinuation. Among patients who underwent ≥2 attempts at levothyroxine discontinuation, subsequent discontinuation attempts were performed at ≥3 months after the last attempt.

Postoperative hypothyroidism was defined as TSH levels of ≥4.20 mIU/L (RR: 0.27–4.20 mIU/L). Successful levothyroxine discontinuation was defined as the patient exhibiting normal TSH levels without thyroid hormone treatment for 2 years after the hemithyroidectomy.

### 2.5. Statistical Analysis

The patients were categorized according to their levothyroxine discontinuation outcomes: (1) maintenance of normal thyroid function or (2) treatment failure with functional decline. We evaluated the associations of successful levothyroxine discontinuation with age, sex, thyroid autoantibodies, timing and frequency of levothyroxine discontinuation, thyroid parenchyma histopathology, and the anteroposterior diameter of the residual thyroid. Continuous and categorical variables were compared using the independent samples* t*-test and chi-square test, as appropriate. The chi-square test and Fisher's exact test were used to compare the factors that influenced the levothyroxine discontinuation outcomes after hemithyroidectomy. Patients were divided into two age groups for some comparisons (<45 years and ≥45 years). The initial discontinuation timing was compared between the treatment success and failure groups using the chi-square test. Continuous variables were reported as mean ± standard deviation. We used SPSS software (version 15.0; SPSS Inc., Chicago, IL, USA) for all analyses, and a* p* value of <0.05 was considered statistically significant.

## 3. Results

Among the 251 patients, lymphocytic thyroiditis was histopathologically confirmed in 43 patients (17.1%), although no patients exhibited Graves' disease or diffuse hyperplasia. The characteristics of the treatment success (*n* = 125, 49.8%; mean age, 42.8 years) and failure (*n* = 126; mean age, 41.9 years) groups are listed in Table 1. The mean follow-up period from the last discontinuation attempt was 34.8 ± 15.9 months (range, 24–68 months). Preoperative TSH was significantly associated with treatment success, although treatment success was not significantly associated with patient age, the anteroposterior diameter of the residual thyroid, preoperative anti-TPO levels, and preoperative anti-TG levels.

The results of the univariate analyses are shown in Table 2. The distributions of age (*p* = 0.7) and sex (*p* = 0.649) were similar between the two groups. Successful levothyroxine discontinuation was significantly associated with the underlying diffuse thyroid disease (*p* = 0.001). In addition, there was a high prevalence of treatment failure (77.8%, 28/36) among cases of non-Hashimoto lymphocytic thyroiditis. There was a significant difference in the preoperative TSH levels between the success and failure groups (*p* < 0.001). When the preoperative TSH levels were ≥2.3 mIU/L, there was a high probability of treatment failure. However, the thyroid autoantibody levels were not significantly different between the two groups (*p* = 0.844). The number of discontinuation attempts was positively correlated with the levothyroxine discontinuation success rate (*p* < 0.001). In the success group, successful levothyroxine discontinuation occurred after the first attempt in 90 patients, after the second attempt in 19 patients, after the third attempt in 2 patients, after the fourth attempt in 5 patients, and after the fifth attempt in 9 patients. In the failure group, 63 patients underwent one discontinuation attempt, 61 patients underwent two discontinuation attempts, and 2 patients underwent three discontinuation attempts. The two groups exhibited similar ages (*p* = 0.516) and anteroposterior diameters of the residual thyroid lobe after the hemithyroidectomy (success, 12.97 ± 2.61 mm; failure, 13.02 ± 2.82 mm;* p* = 0.875). The highest failure rate was observed when the discontinuation was attempted at =3 months after the hemithyroidectomy (*p* < 0.001).

The rate of euthyroidism maintenance was significantly lower for levothyroxine discontinuation at ≤3 months, compared to levothyroxine discontinuation at ≥4 months. The timings for the initial levothyroxine discontinuation after hemithyroidectomy and the frequencies of successful levothyroxine discontinuation are summarized in Table 3. Most patients underwent the initial discontinuation attempt at <36 months after the hemithyroidectomy, although 5 patients (2.0%, 5/251) underwent the first discontinuation attempt at ≥36 months. However, none of these 5 patients achieved successful levothyroxine discontinuation. In the univariate analysis, there was a significant difference in the outcomes for initial discontinuations at ≤3 months or ≥4 months after the hemithyroidectomy.

## 4. Discussion

Among patients with PTMC, hemithyroidectomy has been used for low-risk patients with a single lesion that is restricted to the thyroid gland and no cervical lymph metastasis [6]. Hemithyroidectomy is often the preferred surgical option because thyroid function can be preserved without thyroid hormone replacement, although the prevalence of hypothyroidism after hemithyroidectomy ranges from 10% to 60% [11–13]. This wide disparity might be related to differences in the studies' definitions and follow-up durations [14]. In the present study, 126 patients (50.2%) had hypothyroidism after the hemithyroidectomy, despite having undergone at least one levothyroxine discontinuation attempt. The reason for the high failure rate in the present study is not clear, but it might be associated with the limited number of discontinuation attempts.

The preservation of normal thyroid function is essential for patients who are undergoing hemithyroidectomy, although a single lobe theoretically has enough thyrocytes to maintain normal body function [9]. Numerous studies have also demonstrated that preoperative TSH levels are an important factor that affects hypothyroidism after hemithyroidectomy [10–12, 14]. This is likely because high preoperative TSH levels reflect a reduced thyroid functional reserve and are an important cause of postoperative hypothyroidism [11]. Similarly, the present study demonstrated that preoperative TSH levels of ≥2.3 mIU/L were associated with failed levothyroxine discontinuation. In addition, underlying lymphocytic thyroiditis might influence postoperative hypothyroidism after hemithyroidectomy [12, 15], because lymphocyte infiltration and fibrosis in the thyroid parenchyma can gradually destroy the thyroid gland [16, 17]. In the present study, a higher failure rate was observed among patients with non-Hashimoto lymphocytic thyroiditis, compared to patients with a normal thyroid. Furthermore, the remnant gland's volume and older age might affect hypothyroidism after hemithyroidectomy [18, 19], although we did not observe any significant associations of successful levothyroxine discontinuation with patient age, sex, and the anteroposterior diameter of the residual thyroid gland.

In the present study, successful levothyroxine discontinuation was associated with attempts that were performed at ≥4 months after the surgery, and early discontinuation attempts (at ≤3 months) were associated with a low rate of successful levothyroxine discontinuation. The reason for this relationship remains unclear, but it may be related to the hypothesis that hemithyroidectomy induces the early onset of clinical hypothyroidism [14]. Thus, levothyroxine replacement therapy may be necessary after hemithyroidectomy until thyroid function has been stabilized. However, it is also possible that thyroid function after hemithyroidectomy changes over time, and that the administration of levothyroxine after hemithyroidectomy might delay the recovery of normal thyroid function. Thus, large-scale prospective studies are needed to further evaluate this issue.

There are several limitations in the present study. First, the levothyroxine discontinuation timing was irregular. Second, the mean follow-up period was 34.8 months, which is not sufficient to evaluate long-term success rates. Third, there were a small number of patients (*n* = 5) who initiated levothyroxine discontinuation at ≥36 months after the hemithyroidectomy. Fourth, a small number of patients with underlying Hashimoto thyroiditis (2.8%, 7/251) were included. Finally, we used only the anteroposterior diameter of the residual thyroid gland because of the limited number of US images, and we did not calculate the volume of the residual thyroid gland. Therefore, studies with long-term follow-ups, standardized discontinuation schedules, and larger and more diverse patient populations are needed to address these issues.

In conclusion, the present study's results demonstrate that levothyroxine discontinuation should be initiated at ≥4 months after hemithyroidectomy. In addition, successful levothyroxine discontinuation was associated with underlying lymphocytic thyroiditis, preoperative TSH levels, and the number of discontinuation attempts.

## Figures and Tables

**Table 1:** Individual parameters in the results of levothyroxine discontinuation after hemithyroidectomy.

	Discontinuation of levothyroxine		*p* value
	Success	Failure	
Age	42.81 ± 10.82	41.95 ± 9.98	0.516
Postoperative AP diameter (mm)^*∗*^	10.97 ± 2.61	13.02 ± 2.82	0.875
Preoperative TSH	1.39 ± 0.64	1.88 ± 0.86	<0.001
Preoperative anti-TPO	12.82 ± 47.33	56.96 ± 181.32	0.172
Preoperative anti-TG	27.06 ± 58.80	87.01 ± 183.80	0.128

Note: data are presented as mean ± standard deviation. ^*∗*^Based on ultrasonography; AP, anteroposterior; TSH, thyroid-stimulating hormone; anti-TPO, anti-thyroid peroxidase; anti-TG, anti-thyroglobulin.

**Table 2:** Univariate analysis for the factors between the success and failure of levothyroxine discontinuation after hemithyroidectomy.

	Discontinuation of levothyroxine		*p* value
	Success	Failure	
Age			0.700
<45 years	73	77	
≥45 years	52	49	
Gender			0.649
Male	11	9	
Female	114	117	
Underlying thyroid abnormality			0.001
Normal thyroid	114	94	
Hashimoto thyroiditis	3	4	
Non-Hashimoto lymphocytic thyroiditis	8	28	
Preoperative TSH			<0.001
0.27 ≤ TSH < 2.2	113	84	
2.3 ≤ TSH ≤ 4.2	12	42	
Autoantibody			0.844
Normal	29	31	
Abnormal	96	95	
Number of discontinuation attempts			<0.001
1	90	63	
2	19	61	
3	2	2	
4	5	0	
5	9	0	
Initial discontinuation time			<0.001
≤3 months	38	71	
>4 months	87	55	

Note: data are number of each item.

**Table 3:** Time and results of levothyroxine discontinuation after hemithyroidectomy.

Time of the first levothyroxine discontinuation attempt (month)	Levothyroxine discontinuation		Success rate (%)
	Success	Failure	
Immediately	36	75	32.4
1st	1	2	33.3
2nd	1	2	33.3
3rd	1	4	25
4th	5	0	100
5th	4	2	66.7
6th	4	0	100
7th	3	1	75
8th	2	1	66.7
9th	4	3	57.1
10th	6	8	42.8
11th	4	1	80
12th	9	7	56.2
>1 year	45	20	69.2

Note: data are the number of each item.
